# Transforaminal Percutaneous Endoscopic Lumbar Decompression by Using Rigid Bendable Burr for Lumbar Lateral Recess Stenosis: Technique and Clinical Outcome

**DOI:** 10.1155/2018/2601232

**Published:** 2018-11-26

**Authors:** Shuo Tang, Song Jin, Xiang Liao, Kun Huang, Jiaquan Luo, Tao Zhu

**Affiliations:** ^1^Department of Orthopaedics, The Eighth Affiliated Hospital, Sun Yat-sen University, Shenzhen 517000, China; ^2^Department of Orthopaedics, The 6^th^ Affiliated Hospital of Shenzhen University Health Science Center, Shenzhen 51700, China; ^3^Department of Orthopaedics, Shajing Hospital, Shenzhen 51700, China; ^4^Department of Respiratory Medicine, Second Affiliated Hospital of Chongqing Medical University, Chongqing 400010, China

## Abstract

**Background:**

Open laminectomy has been regarded as the standard surgical procedure for lumbar lateral recess stenosis during the last decades. Although percutaneous endoscopic lumbar decompression has led to successful results comparable with open decompression, its application in LSS with is still challenging and technically demanding. Here, we report the surgical procedure and preliminary clinical outcomes of transforaminal percutaneous endoscopic lumbar decompression (PELD) by using flexible burr for lumbar lateral recess stenosis.

**Method:**

A retrospective study was performed for the patients with lumbar lateral recess stenosis receiving PELD by using flexible burr. The indications of surgery were moderate to severe stenosis, persistent neurological symptoms, and failure of conservative treatment. The patients with mechanical back pain, more than grade I spondylolisthesis, or radiographic signs of instability were not included. Before the operation, the transforaminal epidural lidocaine injections were carried out to make the diagnosis more precise and accurate. Radiologic findings were investigated, and visual analog scale (VAS) for back and leg pain, Oswestry Disability Index, and modified Macnab criteria were analyzed at the different time of preoperation, postoperation, 3 months, 6 months, and 12 months.

**Results:**

The follow-up period was 12 months. The mean VAS scores for back and leg pain immediately improved from 7.9 ± 1.2 to 2.8± 1.3, 2.4 ± 1.0, and 2.3 ± 1.0, respectively. The mean visual analog scale scores (VAS) for back pain and leg pain were significantly improved after PELD. The preoperative ODI dropped from 69.1 ± 7.3 to 25.9 ± 8.7, 25.0± 6.9, and 24.7 ± 6.4, respectively. The final outcome was excellent in 39.6%, good in 47.9%, fair in 8.3%, and poor in 4.17%. 87.5% of excellent-to-good ratio was achieved on the basis of Macnab criteria at postoperative 12 months. The complications were limited to transient postoperative dysesthesia (one case), temporary pain aggravation (six cases), and neck pain during the operation (one case).

**Conclusion:**

This observation suggests that the clinical outcomes of PELD for lateral recess stenosis were excellent or showed good results. This minimally invasive technique would be helpful in choosing a surgical method for lateral recess stenosis.

## 1. Introduction

Lumbar spinal stenosis (LSS) is the most common indication for lumbar spine surgery in elder people. With the steadily rising elderly population, the number of patients suffering low back pain and leg pain who seek medical care for lumbar spinal stenosis (LSS) has also increased. Surgical treatment showed greater benefits for patients with moderate to severe lumbar spinal stenosis compared with conservative treatment [1].

For lateral recess stenosis, the traditional surgical posterior approach generally involves a posterior decompression with foraminotomy and facetectomy, which enlarges the nerve root canalis and can be performed with lumbar fusion [2]. However, posterior laminectomy often leads to the destruction of the stability of the motion segments due to its resection of the lamina, the isthmus, and the intervertebral facet joints; further, this procedure can induce scarring of the epidural space, postoperative back pain, and complications [3, 4]. A paraspinal approach was firstly reported in 1987 [5]. This technique enables direct decompress the foraminal or lateral recess with minimal violation of the facet joint and less postoperative back pain. However, some patients experience postoperative leg pain or dysesthesia due to the excessive manipulation of the dorsal root ganglion [6, 7]. Moreover, a limited field of view in this approach may lead to insufficient decompression.

To solve the problems above, several minimally invasive techniques including endoscopic spine surgery have been developed. At the beginning, the application of endoscopic spine surgery was limited to soft disc herniation [8]. With the evolution of endoscopic techniques and instrumentation, and the indication has been expanded. High-speed endoscopic drills and reamer kits enable the treatment from various types of disc herniation to moderate/severe stenosis. The transforaminal percutaneous endoscopic lumbar decompression (PELD) as minimal invasive techniques has many advantages over open surgery, including less paravertebral muscle injury and postoperative pain, preservation of segment instability, minimizing epidural scarring, and rapid recovery [9].

**Figure 1:**
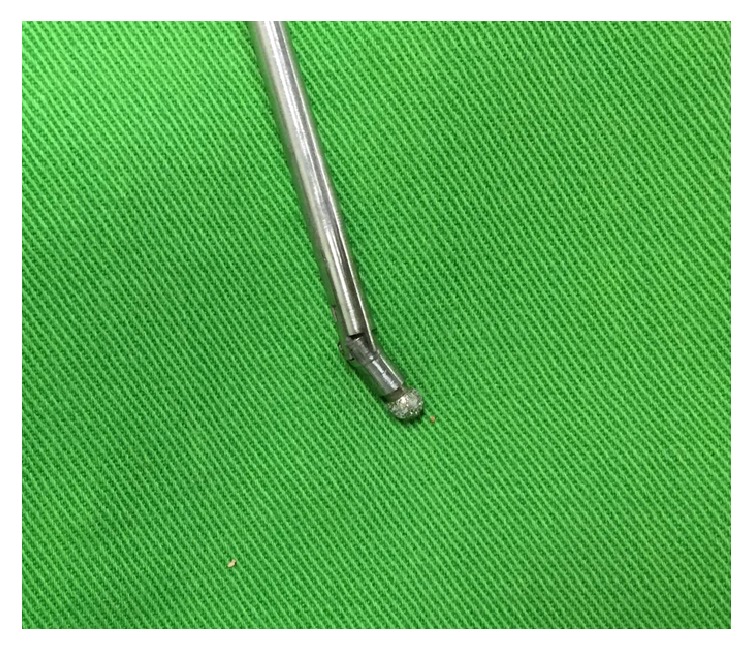
Picture of the rigid bendable burr for decompression. The tips of the burrs can bend 40 degrees.

However, to our knowledge, at present, there are few relevant studies on PELD techniques for the treatment of lateral recess stenosis. A sufficient and precise decompression has been difficult to achieve for hard bony stenosis. Recently, novel rigid bendable high-speed endoscopic burrs (Xishan, Chongqing, China) have been developed and used for the endoscopic spinal surgery. The speed of the burrs can be achieved as high as 20000 rpm/min. The tips of the burrs can bend 40 degrees (Figure 1). The purpose of this clinical series was to describe the PELD technique by using the rigid bendable high-speed endoscopic burrs in the treatment of lumbar lateral recess stenosis and to document the clinical outcomes.

## 2. Materials and Methods

**Figure 2:**
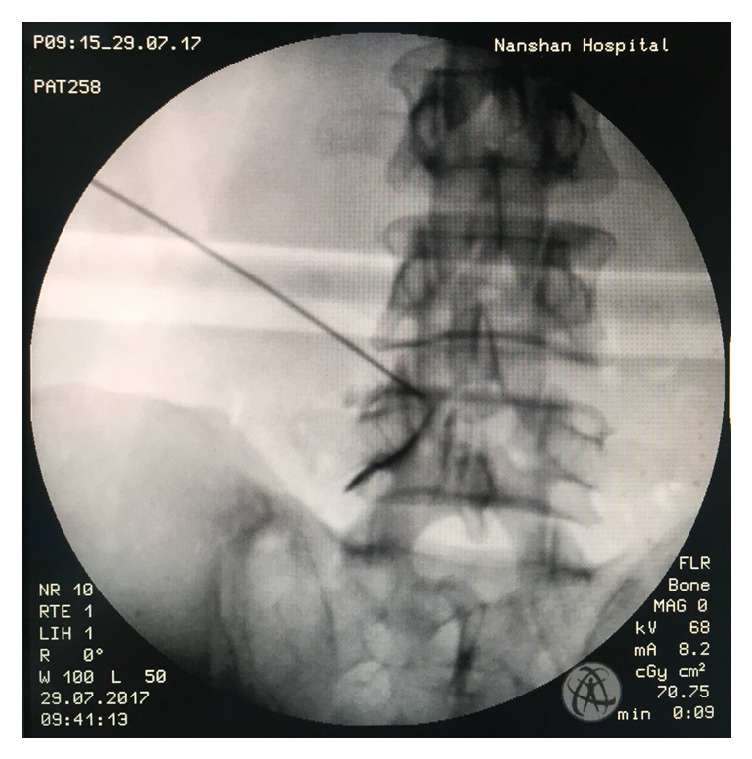
Transforaminal epidural lidocaine injections with Kambin's triangle approach before PELD. A small amount of contrast is used to confirm epidural spread and nerve root.

Totally 48 consecutive patients who were treated between January 2016 and April 2017 by this technique were reviewed retrospectively. Inclusion criteria were as follows: (1) neurogenic claudication or radicular leg pain with (2) moderate to severe lateral recess stenosis shown on cross-sectional MRI or CT scan images, (3) failure of conservative treatment for at least 3 months, and (4) transforaminal epidural lidocaine injections which can relieve the symptom temporarily (Figure 2). Exclusion criteria were as follows: (1) patients with spondylolisthesis grade II or greater; (2) patients who demonstrated frank segmental instability in dynamic radiographs; (3) patients who had mechanical low back pain; the mechanical lower back pain could be defined as pain that prevented the patient from standing or sitting for more than thirty minutes, or induced by posture change; (4) patients who were inoperable due to other medical problems; and (5) coexisting pathological conditions including, infection, acute inflammation, and tumor.

### 2.1. Surgical Technique

**Figure 3:**
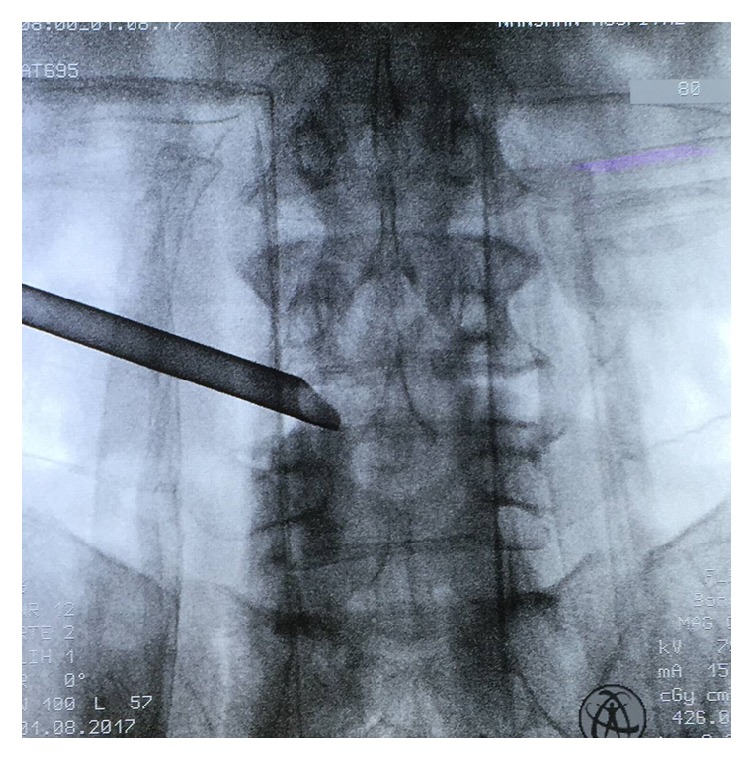
Radiography showing the working cannula has successfully reached the operation area at L4-5 level.

**Figure 4:**
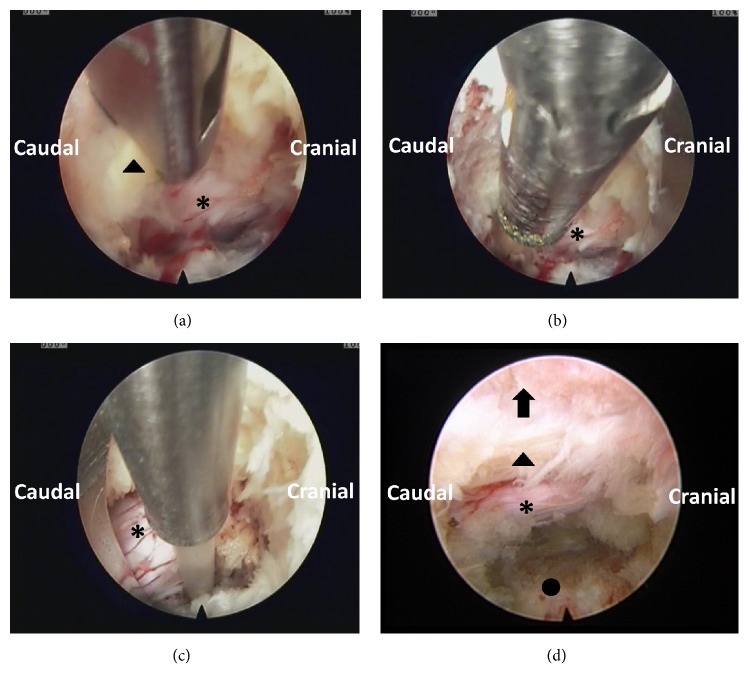
Endoscopic views of the surgical procedure. (a) Wide range of bone resections by using the rigid bendable burr. (b) Removing of the ligamentum flavum and extruded disc by the forceps. (c) Clearing the soft-tissue in the operating field and hemostasis with the help of the bipolar radiofrequency electrocoagulator. (d) Final view of the lateral recess decompression status. Note the free mobilization and release of the nerve root (arrow head means lamina, arrow mean ligamentum flavum, *∗* nerve root, and ○ lateral recess).

All operations were performed under local anesthesia. The patients were placed on a radiolucent orthopedic surgery bed allowing the lumbar spine to be flexed as much as possible to widen the interlaminar space. After confirming the segment, the target point and skin entry point were marked under posterior-anterior and lateral radiographs. The entry point is selected 10 to 14 cm from the midline. Subcutaneous tissue and trajectory tract were infiltrated with 5-10 ml 1% lidocaine at the target level after a routine disinfection procedure. Following this, an 18-gauge needle was inserted with the guidance of C-arm. The needle tip was positioned at one point of the medial-to-lateral pedicular line on the anteroposterior fluoroscopic projection and at the posterior vertebral line on the lateral projection. In the process of puncture, the target point of the needle tip was not the intradiscal portion, but the surface of the facet joint [7], because the main target of this procedure was the foraminal nerve root entrapment by thickened foraminal ligaments and bony stenosis, not the herniated disc fragments. This is a little different from the usual transforaminal endoscopic discectomy technique. Another 10 mL 0.75% lidocaine was injected for further anesthesia, and a guide wire was inserted as the direction of the needle. After that, serial dilation and trephine channel were inserted as the direction of guide wire. Finally, a working channel was inserted into the intervertebral foramen. Next, an endoscope (Joimax, GmbH, Karlsruhe, Germany) was inserted through the channel (Figure 3). Surgical procedures were performed in the following sequence: (1) drilling of bony structures around the ligamentum flavum. The hypertrophied superior facet was firstly undercut. After that, the working cannula could be engaged with the widened foramen [7]. (2) We carried out a carefully exploration, and then the intraforaminal structures including the shoulder osteophyte, ligamentum flavum, perineural fat covering the transverse nerve root, and lumbar disc appeared clearly. Bone debris and tenacious ligaments were removed by using endoscopic burr and punches [7]. When we rotated the axis of burr, a wider range of bone resections under limited endoscopic visual field will be feasible. We often set the speed as 15000-20000 rpm/min. (3) Soft tissues and redundant disc can be coagulated with the help of the bipolar radiofrequency electrocoagulator (Gaotong, Xian, China). After removing the ligamentum flavum, bony structures around, and extruded disc, the lateral recess was enlarged. The end point of the procedure is free mobilization and release of the nerve root (Figure 4). After adequate hemostasis, the working channel and endoscope were withdrawn, and the skin was closed with 2-0 nylon sutures.

### 2.2. Outcome Measures

**Figure 5:**
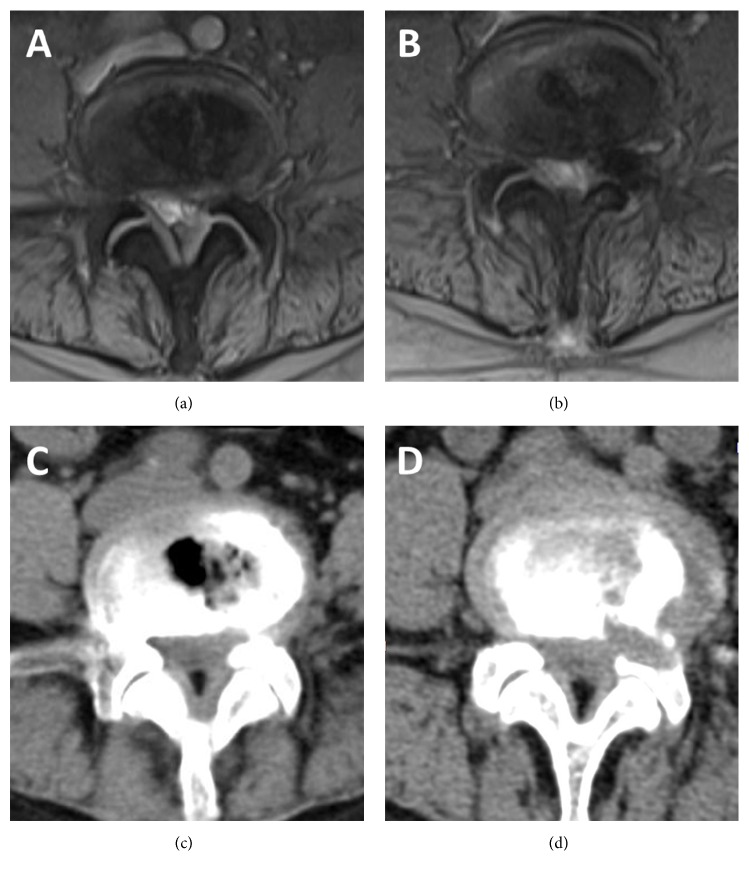
Illustrated case of a female patient. (a, c) Preoperative MR (a) and CT (c) images showing severe lateral recess stenosis at the left L4-5 level. (b, d) Postoperative MR (b) and CT (d) images showing lateral recess decompression after PELD.

MRI and CT scan were done at the second postoperative day after the surgery (Figure 5). The outcomes with a visual analog scale (VAS), Oswestry Disability Index (ODI), Macnab criteria, and complication rate were analyzed preoperatively and repeated in the immediate postoperative period and also at months 3, 6, and 12 after surgery.

## 3. Results

**Figure 6:**
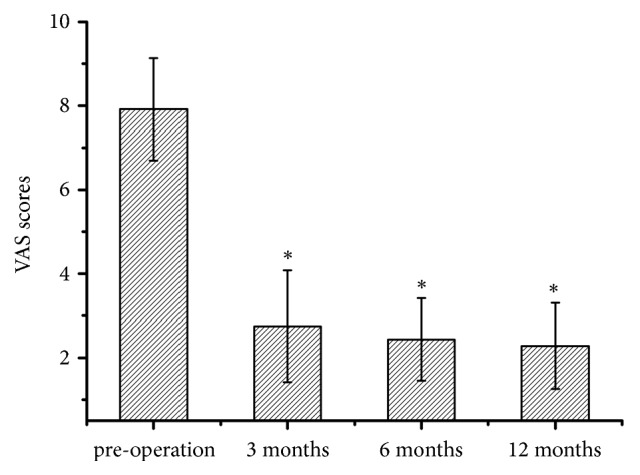
VAS score of leg pain before operation and at each time point postoperation. *∗*Compared with preoperative, p<0.05.

**Figure 7:**
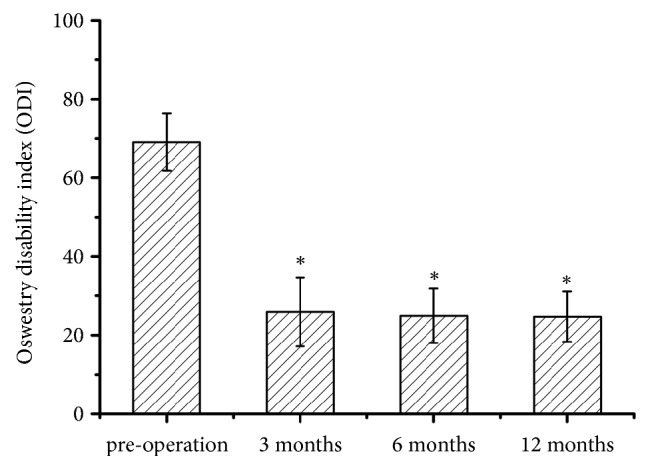
ODI before operation and at each time point postoperation. *∗*Compared with preoperative, p<0.05.

There were 20 men and 28 women (48 patients) with a mean age of 69.2 years (45-81 years). Totally 61 levels were decompressed in 48 patients. The L4-5 was involved most commonly (50 cases), followed by L3-4 (6 cases), L2-3 (3 cases), and L5-S1 (2 cases). Thirty-five patients (72.9%) had one-level and thirteen (27.1%) had two-level decompression. The overall VAS score was improved from 7.9 ± 1.2 to 2.8± 1.3, 2.4 ± 1.0, and 2.3 ± 1.0 (Figure 6), respectively. ODI (%) was improved from 69.1 ± 7.3 to 25.9 ± 8.7, 25.0± 6.9, and 24.7 ± 6.4 (Figure 7). At the final follow-up review, the modified Macnab criteria were rated as follows: excellent in 19 patients (39.6%), good in 23 patients (47.9%), fair in 4 patients (8.3%), and poor in 2 patients (4.17%). The satisfactory rate was 87.5%. Two patients underwent a second surgery for additional decompression and fusion. Complications were limited to transient postoperative dysesthesia (one case), temporary pain aggravation (six cases), and neck pain during the operation (one case). Infection did not happen.

## 4. Discussion

Spinal degeneration is a natural aging process that occurs as we grow older. With the increasing mean length of human life, the prevalence of spinal stenosis is expected to increase in the elderly population [10]. Major causative factors in LSS are hypertrophied facet joints, osteophyte formation, disc protrusion, and hypertrophy of the ligamentum flavum, in which the spinal canal becomes narrow and symptoms arise from mechanical compression of the nerve root and/or cauda equine or vascular insufficiency leading to blood flow loss in the nervous system of the lumbar spine [11]. Lumbar spinal stenosis can be classified into three categories according to pathological zone as follows: central stenosis, lateral recess stenosis, and foraminal stenosis [10]. Central stenosis is defined as a change in the shape of the central canal and dural sac to flattened ovals or triangles, with obliteration of cerebrospinal fluid on the axial images. Lateral stenosis is defined when there was a loss of tail of fat shadow in the sagittal view, trefoil narrowing of the lateral recess, or angular pinch-like encroachment of the lateral margin of the canal with obliteration of cerebrospinal fluid surrounding the nerve root in the axial view. Foraminal stenosis is defined as the compression of the exiting nerve root by various pathologies such as hypertrophied bone and ligament, osteophytes, and/or redundant disc [12]. Traditionally, lumbar spinal stenosis is treated with an open, decompressive laminectomy with or without facetectomy.

Recently, several less invasive techniques have been introduced over the past decade including the percutaneous endoscopic technique. At the beginning, the main indication for percutaneous endoscopic spine surgery for lumbar disc disease has been soft disc herniation. The development of new surgical access and corresponding instruments and burs has expanded the indication spectrum for percutaneous endoscopic operations on the lumbar spine [13, 14]. With the emergence of endoscopic burrs, the surgical indications of spinal endoscopy are expanded and may include lumbar spinal stenosis.

One of the most important concepts in terms of endoscopic spine surgical approaches is understanding the pathologic neuroforaminal anatomy in terms of both disc pathology and facet changes. The endoscopic approach is determined according to the classification of the lumbar stenosis. For central stenosis, bilateral decompression may be necessary. Posterior biportal unilateral approach or uniportal approach for bilateral decompression was often chosen [15]. For lateral recess stenosis, both posterior interlaminar and transforaminal approaches are appropriate. It depends on the experience of the surgeon and the radiographic evaluation. Surgeons who are familiar with the conventional microdiscectomy or microendoscopic discectomy technique may prefer interlaminar approaches. Surgeons who are trained and have experience with transforaminal PELD may prefer transforaminal approach. For the foraminal stenosis, the transforaminal approach and foraminoplasty are appropriate [15]. In this series, we chose the transforaminal approach. The most important advantage of this approach is that the surgeon can directly access the spinal canal and disc space without any significant neurovascular or bone barriers [16].

Because of minimal invasiveness, diagnosis of the altered anatomy and physiology is critical and must be precise and accurate. Pao has reported two cases of wrong level surgeries [17]. Furthermore, 35% of patients with LSS had more than one level of moderate to severe stenosis according to radiographs; therefore, single-level laminectomy is a risk factor for poor outcome [18, 19]. For satisfactory outcome, we preformed transforaminal epidural lidocaine injections (TFELIs) before the PLED operation. Transforaminal epidural steroid injections (TFESIs) are often used to treat lumbar foraminal stenosis. Local anaesthetics and steroid were injected into the anterior extradural space and around nerve root, which was expected to contribute to pain reduction by interrupting the synthesis of prostaglandins, blocking and controlling edema around the nerve root [20]. In this series, the injection did not contain steroid, and only 2% lidocaine (0.3 ml) was injected. 0.3ml lidocaine would diffuse in local area. It can help us to identify the diagnosis and predict the effects of the operation. If the patients obtained satisfactory pain relief and VAS scores decrease more than 50%, we concluded the operation would be helpful. If not, the operation would not be performed.

Precise and sufficient decompressions are the two key factors to the treatment of lumbar spinal stenosis. Traditionally, decompression plus fusion surgery has been regarded as the standard surgical procedure for LSS during the last decades [21, 22]. However, as early as 1996, Kambin P. et al. reported the technique of transforaminal endoscopic decompression for lateral recess stenosis [23]. Forsth et al. presented convincing data that, among patients with lumbar spinal stenosis, with or without degenerative spondylolisthesis, decompression surgery alone has the comparable clinical outcomes with decompression plus fusion surgery at 2 years and 5 years. In 2016, Epstein reviewed Weinstein's randomized controlled SPORT trial data from 13 sites involving 2,500 patients including degenerative spondylolisthesis and lumbar spinal stenosis. Patients with degenerative spondylolisthesis and associated LSS undergoing decompression alone versus noninstrumented versus instrumented fusion had comparable results [24, 25]. Postacchini reported satisfactory outcome from several studies of patients with lateral stenosis after laminotomy of 79–93% [21]. In this series, the outcome as satisfactory was 87.5%.The satisfactory outcome can ascribe to the TFELIs before operation and the application of rigid bendable burrs, which are helpful for precise and sufficient lateral recess decompression. Compared with original unbendable burr, the wider range of bone resections under limited endoscopic visual field will be feasible. During the operation, the bending angle can be adjusted from 0 to 40 degrees according to the requirement of decompression, which made the decompression more sufficient.

According to published literatures, potential complications of endoscopic operation include nerve root injury, durotomy, infection, retroperitoneal cavity injury, cauda equine injury, great vessel injury, muscular hematoma, and epidural hematoma [26, 27]. In our series, one patient complained of severe posterior neck pain and weakness in both lower limbs. This symptom was somehow related to the increased epidural pressure (EP) and intracranial pressure (ICP) caused by the massive use of irrigation fluid during the procedure [28]. As we know, high ICP is known to lead to serious complications, such as visual loss due to retinal hemorrhage and even loss of consciousness [29]. In the occurrence of posterior neck pain, we stopped the procedure and drain out the irrigation fluid of the epidural space through the working channel. Until the pain relieved for 10 minutes, we decreased the speed of irrigation fluid and continued the procedure. To avoid this complication, the use of a saline irrigation pump to controlled pumping pressure and avoid overincreased epidural hydrostatic pressure is important. The pressure of saline irrigation pump is kept between twenty-five and thirty mmHg, depending on the patient's condition for lumbar surgery [30]. Six patients experienced temporary pain aggravation. This may be caused by the nerve violation during the procedure. They were treated with nonsteroidal anti-inflammatory drugs and methylprednisolone for several days according to the symptom. One patient experienced transient postoperative dysesthesia. This symptom subsided within 3 months. This can be ascribed to excessive manipulation or irritation of the dorsal root ganglion during lateral recess decompression. Dural tearing does not occur, since the ligamentum flavum is kept as protective barrier for the dura mater until completion of the bony procedure. Furthermore, we can get a good visualization of the dural sac and nerve root with the help of endoscope. Two of the 48 patients underwent open fusion surgery due to incomplete decompression. To avoid the secondary operation, sufficient decompression is crucial. The surgeon should be patient and careful during the procedure. Overall, the complication rate of endoscope in this series was similar to those of transforaminal endoscopic surgery and lower than microscope-assisted procedures [31, 32].

We obtained satisfactory results according in this preliminary study; however, there are some limitations. Firstly, because the initial benefits of minimally invasive decompression may deteriorate over time, the longer follow-up times is needed to obtain more accurate results and conclude the long-term benefits. Secondly, there is no control group for comparison. Further, longer term and randomized controlled trial would be conducted. We should pay more attention on duration of symptoms relief, the risk of postoperative instability, and the incidence of reoperation.

## 5. Conclusions

The preliminary results suggest that the clinical outcomes of PELD combined with rigid bendable burr for lumbar lateral recess stenosis were safe and effective. However, a long-term randomized controlled and more detailed trial would be needed for more accurate results of PELD technique.

## Data Availability

The authors have full control of all primary data and they agree to provide them upon request.
